# Solution Casting Effect of PMMA-Based Polymer Electrolyte on the Performances of Solid-State Electrochromic Devices

**DOI:** 10.3390/polym17010099

**Published:** 2025-01-02

**Authors:** Abdelrahman Hamed Ebrahem Abdelhamed, Gregory Soon How Thien, Chu-Liang Lee, Benedict Wen-Cheun Au, Kar Ban Tan, H. C. Ananda Murthy, Kah-Yoong Chan

**Affiliations:** 1 Centre for Advanced Devices and Systems, Faculty of Engineering, Multimedia University, Persiaran Multimedia, Cyberjaya 63100, Selangor, Malaysia; abdelrahmanhamed.eg@gmail.com (A.H.E.A.); gregory@mmu.edu.my (G.S.H.T.); cllee@mmu.edu.my (C.-L.L.); benedictau@gmail.com (B.W.-C.A.); 2 Sri Desa International Secondary School, Taman Desa 58100, Kuala Lumpur, Malaysia; 3 Department of Chemistry, Faculty of Science, Universiti Putra Malaysia, Serdang 43400, Selangor, Malaysia; tankarban@upm.edu.my; 4 Department of Applied Sciences, Papua New Guinea University of Technology, Lae 411, Morobe Province, Papua New Guinea; anandkps350@gmail.com

**Keywords:** electrochromic device (ECD), PMMA, solid polymer electrolyte (SPE), sol gel, spin coating, solution casting, WO_3_, smart glass

## Abstract

Electrochromic devices (ECDs) are devices that change their optical properties in response to a low applied voltage. These devices typically consist of an electrochromic layer, a transparent conducting substrate, and an electrolyte. The advancement in solid-state ECDs has been driven by the need for improved durability, optical performance, and energy efficiency. In this study, we investigate varying the temperature to the casting solution for polymethylmethacrylate (PMMA)-based electrolytes for solid-state ECDs with a structure of glass/ITO/WO_3_/PMMA electrolyte/ITO/glass. The electrochromic layer, composed of WO_3_, was deposited using the sol-gel method, while the electrolyte, comprising lithium perchlorate (LiClO_4_) in propylene carbonate (PC) with PMMA, was prepared via solution casting. Various electrolyte samples were heated at different temperatures of 25, 40, 60, 80, and 100 °C to analyze the impact on the devices’ performance. Our findings indicate that the devices with electrolytes at 25 °C exhibited superior anodic and cathodic diffusion. An increase in heating temperature corresponded with an increase in switching time. Notably, the sample heated at higher temperatures (60, 80, and 100 °C) demonstrated exceptional cycle stability. Nevertheless, samples with higher temperatures displayed a decrease in optical modulation. Additionally, the 100 °C sample exhibited the highest coloration efficiency compared to other samples at lower temperatures. This research highlights the potential of varying the temperature of solution casting on PMMA-based electrolytes in optimizing the performance of solid-state ECDs, particularly regarding coloration efficiency and durability.

## 1. Introduction

As global energy consumption continues to rise, reducing dependency on fossil fuels and minimizing the impact of energy production on the environment are becoming key challenges. The development of energy-efficient technologies has significantly increased, especially in the field of building management [1]. Smart glass technologies have emerged as a leading technology in the pursuit of energy efficiency and sustainability, especially in the building and automotive sectors. These glasses, which can dynamically adjust their optical properties in response to external stimuli, can decrease the demand for lighting and HVAC systems in a building by regulating the amount of solar heat and light entering, thereby contributing to less energy consumption [2]. Existing studies reported that these devices can reduce energy demands for cooling systems by up to 25–38%, depending on the climate and application [3].

Several smart glass technologies have been invented in recent years with each having a different working mechanism, notably electrochromic (EC) glass [4], thermochromic glass [5], photochromic glass [6], and suspended particle devices (SPD) [7], as well as Polymer-Dispersed Liquid Crystal (PDLC) along with others [8]. For instance, thermochromic glass changes its optical properties based on temperature, while photochromic glass reacts to light intensity [5]. Additionally, SPDs use suspended particles to adjust optical transparency in response to an electric field [7]. EC glasses (or devices) are among the most promising technologies in achieving energy savings in buildings due to their ability to adjust transparency (or color) when a low electric voltage is applied [9]. These devices enable the dynamic control of solar light and heat penetration into a building, contributing to temperature regulation and reduced energy usage for lighting and cooling [10].

The working principle of EC devices (ECDs) involves a reversible redox reaction facilitated by the movement of ions between layers in response to an applied voltage. Typically, an ECD consists of a multilayer structure comprising a transparent conducting oxide (TCO), an EC layer (such as tungsten oxide [WO_3_]), an electrolyte, and a counter electrode. When a small voltage is applied to these devices, ions from the electrolyte move into the EC layer, causing a change in its optical properties—switching between a transparent and colored state. This phenomenon, which is known as electrochromism, is essential for ECDs like smart windows, displays, and mirrors [11].

Electrolytes are essential for the operation of ECDs where they facilitate ion transportation between the electrodes. High ionic conductivity, chemical stability, and the capacity to withstand several cycles of coloration (ion insertion) and bleaching (ion extraction) are all criteria of an ideal electrolyte [4]. Electrolytes can be in different forms—liquid, gel, or solid—depending on the device structure and performance requirements. The choice of electrolyte affects the overall device efficiency, cycling stability, and durability. For instance, liquid electrolytes have high ionic conductivity and good optical modulation, but they are less suitable for long-term usage due to evaporation and leakage issues [12]. Conversely, gel electrolytes provide high ionic mobility and enhanced mechanical stability, though their ionic conductivity is often lower than that of liquid electrolytes [13]. Solid polymer electrolytes (SPEs) are known for their exceptional mechanical stability and durability, particularly in large-area devices [14]. However, their ionic conductivity is often lower, especially when operating at room temperature. Solid electrolytes are essential for realizing an all-solid-state ECD [15].

Several fabrication methods were developed to fabricate a solid electrolyte, including solution (or solvent) casting [16], in situ polymerization [17], and magnetron sputtering [18]. These methods aim to produce highly efficient electrolytes with enhanced performance, especially in terms of ionic conductivity and mechanical stability. Among these, the solution casting method is particularly promising due to its ability to produce thin, consistent films of solid polymer electrolytes, with good ionic conductivity and high cycling stability. This method is preferred for its simplicity and scalability, making it suitable for both laboratory and industrial applications [19].

The solution casting method involves dissolving a polymer and salt in a solvent, casting the solution onto a substrate, and evaporating the solvent to form a thin electrolyte film [19,20]. The quality of the resulting film can be controlled by adjusting the concentration of the polymer, salt, and solvent, as well as the casting and drying conditions [21]. Despite the advantages of the solution casting method, there is limited research on the utilization of this method for solid polymer electrolytes in ECDs. Additionally, further research is required to optimize the processing conditions to ensure uniformity and consistency in large-area applications.

The heating temperature utilized during solvent evaporation (solution drying) is a significant component of the solution casting method. Varying the heating temperature can significantly affect the crystallinity, porosity, and ionic conductivity of the resulting electrolyte film [21,22,23,24]. However, there is still limited understanding of how specific temperature variations impact the long-term performance of ECDs. Research is needed to optimize the thermal treatment process to achieve the ideal balance between the electrolyte structure and function. As far as the authors are mostly aware, there has been no research conducted on the effect of varying heating temperatures on the solution casting for solid polymer electrolytes for ECD applications. Therefore, this study proposed a novel strategy to investigate how varying the heating temperature during the solution casting of PMMA-based electrolytes can optimize the performance of ECDs.

In this study, the effects of varying heating temperatures for the solution casting for a PMMA-based electrolyte for ECD were investigated. The device structure utilized was based on the lamination scheme with a structure of glass/ITO/WO_3_/PMMA electrolyte/ITO/glass. Different ECD samples were fabricated with different solution casting temperatures, namely 25, 40, 60, 80, and 100 °C. The measurements for all devices were conducted at room temperature, with each device allowed to stabilize at room temperature before data collection.

## 2. Materials and Methods

### 2.1. Materials

Tungsten hexachloride (WCl_6_), 30% hydrogen peroxide (H_2_O_2_), lithium perchlorate (LiClO_4_), propylene carbonate (PC), acetonitrile (ACN), and polymethylmethacrylate (PMMA) were purchased from Sigma-Aldrich, Rockville, MD, USA. Glacial acetic acid (HoAc [CH_3_COOH]) was obtained from the BASF Chemical Company, Ludwigshafen, Germany. Subsequently, absolute ethanol (EtOH, C_2_H_6_O) was purchased from Merck, NJ, USA. The ITO was obtained from the Han Xin Industry Co., Ltd., Shanghai, China. All chemicals utilized in this study were of analytical grade and were used as received without any further purification.

### 2.2. Methods

#### 2.2.1. Electrochromic Layer Preparation

The WO_3_ thin film solution was prepared via a sol–gel route adopted from the authors’ previous study [25]. In brief, a 20 mL solution with EtOH as the solvent was prepared with concentrations of 0.2, 1.74, and 0.98 M for WCl_6_, HoAc, and H_2_O_2_, respectively. Firstly, WCl_6_, HoAc, and EtOH were mixed and stirred in a glass beaker for 30 min on medium speed using a magnetic stirrer hotplate. Next, H_2_O_2_ was added to the mixture and left to stir for another 30 min. Following that, the solution was heated at 40 °C while stirring for an additional 2 h. Finally, the solution was aged for a minimum of 24 h before deposition.

**Figure 1:**
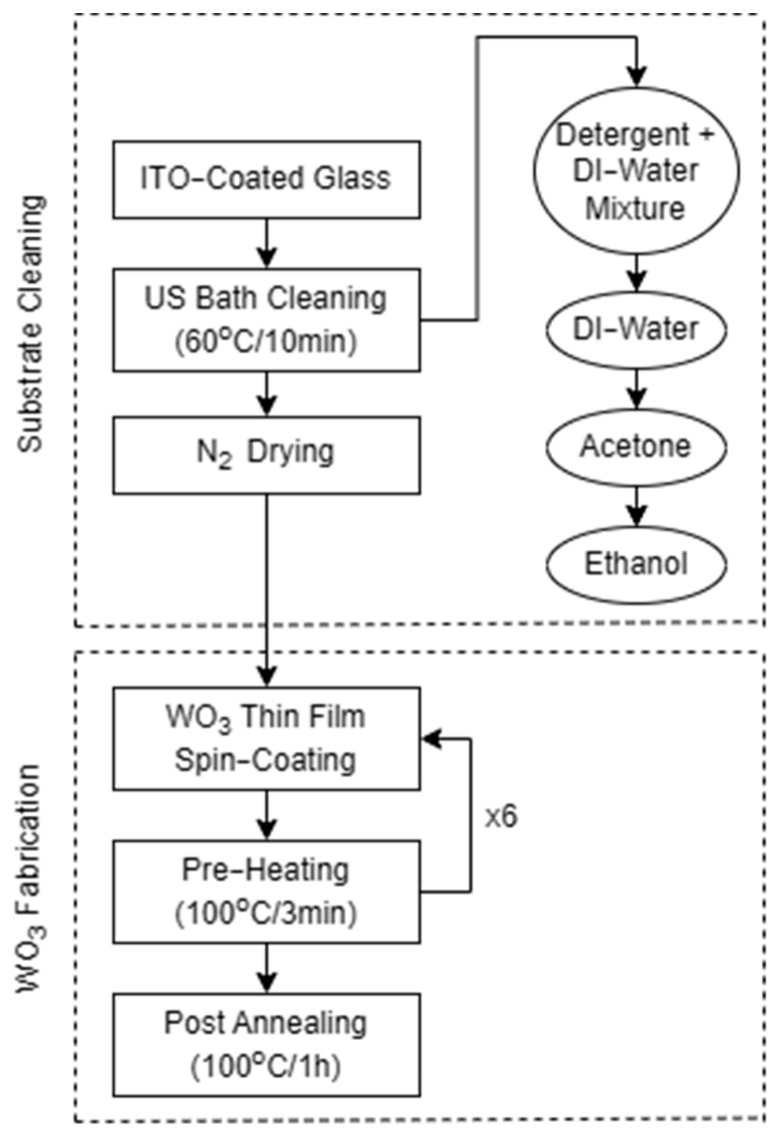
WO_3_Thin Film Fabrication Process.

The WO_3_ electrochromic layer was fabricated using the sol–gel spin-coating method whereas ITO glass with dimensions of 25 × 25 mm was used as the substrate. Prior to deposition, the substrate was cleaned with 30% detergent and a DI-water mixture placed in an ultrasonic bath at 60 °C for 10 min followed by rinsing with DI-water and additional cleaning with acetone and ethanol in ultrasonic bath for 10 min at 60 °C, respectively. After the substrates were cleaned, they were dried using a N_2_ spray gun to remove any excess solvents. Following that, the substrate was masked on one edge using a Kapton tape leaving an unmasked area of 25 × 15 mm. Subsequently, the WO_3_ solution was deposited and spin-coated on the substrate at 3000 rpm for 30 s followed by heating on the hotplate at 100 °C. The spin-coating and heating processes were repeated seven times to obtain a WO_3_ thin film of 330 nm thickness (see Figure 1). Finally, the substrates were annealed in the furnace at 100 °C for 1 h with ramping up and down of 30 min each.

#### 2.2.2. Electrolyte Preparation

To prepare the electrolyte, a 25 mL solution with ACN as the solvent was prepared with a mixture of LiClO_4_, PC, and PMMA with concentrations of 1 M (2.6598 g), 50% *v*/*v* (12.5 mL), and 28% *w*/*v* (7 g), respectively. All chemicals were mixed in a glass beaker and covered with parafilm and stirred on a medium speed using a magnetic stirrer for 24 h. Next, the mixture was placed in the oven for 1 h at 90 °C to get a very viscous gel-like solution. The final electrolyte solution can be used directly without ageing.

#### 2.2.3. ECD Fabrication

**Figure 2:**
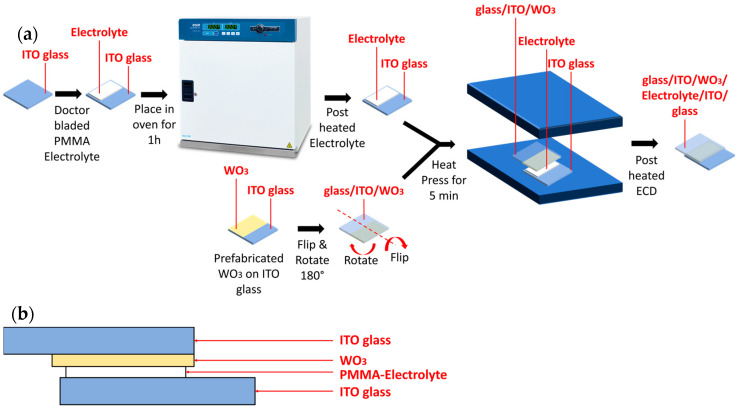
(**a**) Illustration of the ECD fabrication process, highlighting the steps involved in assembling the glass/ITO/WO_3_/electrolyte/ITO/glass structure. (**b**) Final assembled full ECD device. Note that EIS devices follow the same fabrication process but are structured as glass/ITO/electrolyte/ITO/glass without the WO_3_ layer.

In this study, five ECDs were fabricated with the structure of glass/ITO/WO_3_/electrolyte/ITO/glass, while an additional five devices were assembled for electric impedance spectroscopy (EIS) characterization with the structure of glass/ITO/electrolyte/ITO/glass. For each varying temperature, one ECD and one EIS device were used for measurements. In the ECD structure, the electrolyte was doctor-bladed on a pre-cleaned ITO glass substrate after masking the edges and leaving an active area of 22 × 12 mm and a spacing of 0.52 mm using Kapton tape. Next, substrates were placed in the oven separately at varying temperatures (25, 40, 60, 80, and 100 °C) for 1h. Following that, the prefabricated WO_3_ samples were heat-pressed on the glass/ITO/electrolyte samples each at the corresponding temperature for 5 min (25 °C oven → heat-pressed at 25 °C, etc.). For the EIS structure, the assembly steps were the same as in the ECD structure except that a pre-cleaned ITO glass substrate was heat-pressed on the glass/ITO/electrolyte piece instead. Figure 2a,b illustrate the fabrication process and final ECD, respectively.

## 3. Results

### 3.1. Electrochemical Analysis

#### 3.1.1. Cyclic Voltammetry

**Figure 3:**
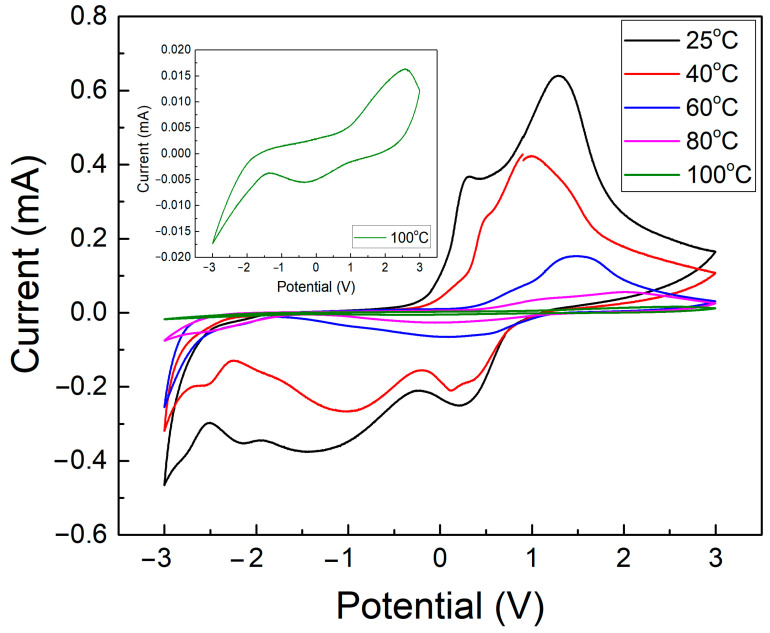
The CV graphs for all samples at different temperatures for the electrolyte used in ECD with the glass/ITO/WO_3_/electrolyte/ITO/glass structure.

Cyclic voltammetry (CV) was conducted for all samples to investigate the reversibility and diffusivity of the fabricated samples. The CV was subjected to a scan rate of 0.1 V s^−1^ and upper and lower voltages of 3 V and −3 V, respectively. Figure 3 shows the voltammograms of all samples. From Figure 3, it can be observed that all samples show a reversible tendency indicating that the ions in the electrolyte layer were inserted and extracted in the EC layer although the reversibility degree decreases as the temperature increases. The peak maximum in the CV curves corresponds to the voltage at which the maximum rate of ion insertion or extraction occurs, indicating the point of highest electrochemical activity. The observed reversible peaks demonstrate ion intercalation and deintercalation in the electrochromic layer. However, as the temperature increases, the peak current decreases, suggesting a reduction in the efficiency of ion diffusion and charge transfer. This trend indicates that higher temperatures affect the electrolyte’s electrochemical performance by altering the number of intercalated and deintercalated charges.

The anodic and cathodic current peaks of the CV voltammograms can be used to determine the ion diffusivity of the samples using the Randles–Sevcik equation [26].

The equation is used to compute the diffusion coefficient (*D*) values for the inserted and extracted ions during the anodic and cathodic cycles, as shown in the following:

(1) Ip=2.687×105n32.A.CD.v12

where *I_p_* is the peak current in amperes, *n* is the number of electrons in the redox cycle (*n* = 1), *A* is the area of the effective area of the WO_3_ thin films (*A* = 2.64 cm^2^), *C* is the molar concentration of the electrolyte (*C* = 1M), *D* is the diffusion coefficient, and *v* is the scan rate used in the CV measurement (*v* = 0.1 V s^−1^).

**Figure 4:**
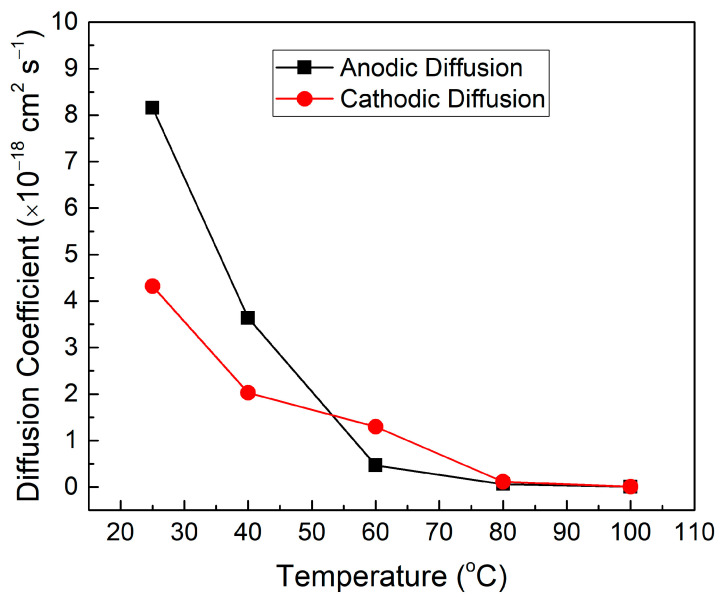
The *D* (anodic and cathodic) for all samples at different temperatures for the electrolyte in the glass/ITO/WO_3_/electrolyte/ITO/glass structure.

Figure 4 demonstrates the *D* for all samples during the anodic and cathodic cycles. From the graph, it can be discerned that the *D* in both anodic and cathodic cycles decrease as the temperature increases.

Table 1 shows the numerical values for the anodic (*D_a_*) and cathodic (*D_c_*) diffusion coefficients for all samples. As observed from Table 1, the 25 °C sample shows the highest *D_a_* and *D_c_* with values of 8.15 × 10^−18^ cm^2^ s^−1^ and 4.32 × 10^−18^ cm^2^ s^−1^, respectively. Alternatively, the 100 °C sample displayed the lowest *D_a_* and *D_c_* with values of 0.0054 × 10^−18^ cm^2^ s^−1^ and 0.0060 × 10^−18^ cm^2^ s^−1^, respectively. The decrease in the *D* values indicates that the ion diffusivity or the ion kinetic response in the electrolyte layer decreases as the temperature increases. This decrease can be due to the difficulty of the ion migration from and to the electrolyte layer because of the increased crystallinity of the electrolyte layer where it hinders the movement of the ions as the temperature increases [27].

#### 3.1.2. Chronoamperometry (CA)

**Figure 5:**
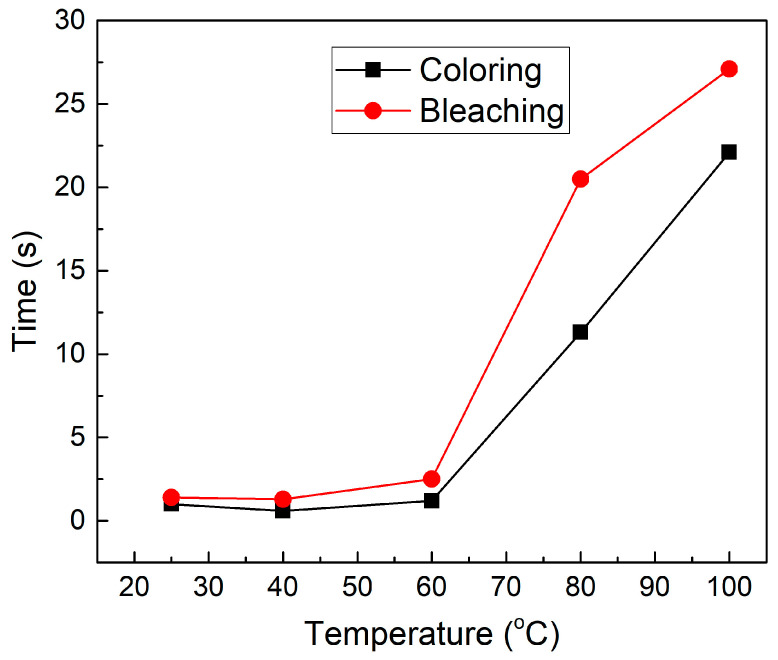
Colouring and bleaching times for all samples at different electrolyte fabrication temperatures in the glass/ITO/WO_3_/electrolyte/ITO/glass structure.

Chronoamperometry (CA) was applied to all samples to discern the switching time and later calculating the coloration efficiency (Transmittance Section) of the fabricated samples. The CA was operated at a duration of 100 s and 60 s with voltages of −3 V and 3 V for the cathodic and anodic cycles, respectively. Figure 5 represents the switching time graph for all samples at 90% of the current evolution of the cathodic and anodic cycles in the CA analysis.

As observed in Figure 5, the coloration and bleaching times increase as the temperature increases. From Table 2, the lowest coloration and bleaching times belong to the 25 and 40 °C samples with values of 1 s/1.4 s and 0.6 s/1.3 s, respectively. The 100 °C sample reported the highest coloration and bleaching times with a value of 22.1 s and 27.1 s, respectively. The increase in the coloration and bleaching times as temperatures increase can be due to the increase in the polymer electrolyte hardness, which inhibits the movement of the ions from and into the electrolyte layer; this supports the previous argument. The faster coloration time compared to the bleaching time can be related to the higher ionic conductivity in the colored state compared to the bleaching state [28].

### 3.2. Transmittance

Transmittance analysis was investigated for all samples using ultraviolet-visible (UV-Vis) spectroscopy. The transmittance data presented emphasize the 300–900 nm range, which includes both the UV and near-infrared (NIR) regions. However, the primary analysis in this study focuses on the visible spectrum (380–780 nm), with the UV and NIR regions mentioned for a more comprehensive understanding of the overall spectral behavior of ECDs.

**Figure 6:**
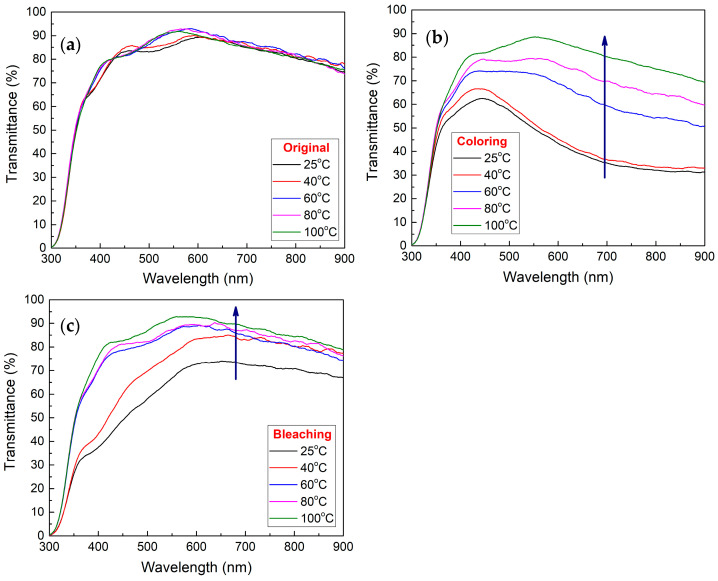
Transmittance images of samples showing: (**a**) original state; (**b**) coloring state, and; (**c**) bleaching state for the glass/ITO/WO_3_/electrolyte/ITO/glass structure.

The transmittance graph was recorded for the fresh samples before any characterization (Original transmittance), and when applying a negative (−ve) potential (Coloring transmittance, −3 V) and a positive (+ve) potential (Bleaching transmittance, +3 V). Figure 6 shows the transmittance graphs for all samples in original (a), bleaching (b), and coloring (c) transmittances. From Figure 6, it can be observed that all samples have a good transmittance prior to any use in the original transmittance graph (Figure 6a) specifically in the visible region and a slight blocking for infrared radiation in the NIR region. In Figure 6b,c, coloring and bleaching showed a tendency to increase with the increase in temperature. For the coloring, this can be due to the limited charges inserted from the electrolyte owing to the increase in crystallinity of the polymer matrix as the temperature increases.

The optical density (*OD*) and optical modulation (*OM*) are calculated for all samples at the 633 nm wavelength using Equations (1) and (2), respectively:

(2) $$\Delta OD=\log\frac{T_{b}}{T_{c}}$$

(3) $$OM=T_{b}-T_{c}$$

where Δ*OD* represents the optical density change, *OM* is the optical modulation, *T_c_* symbolizes the colored state transmittance, and *T_b_* represents the transmittance in the bleached state.

**Figure 7:**
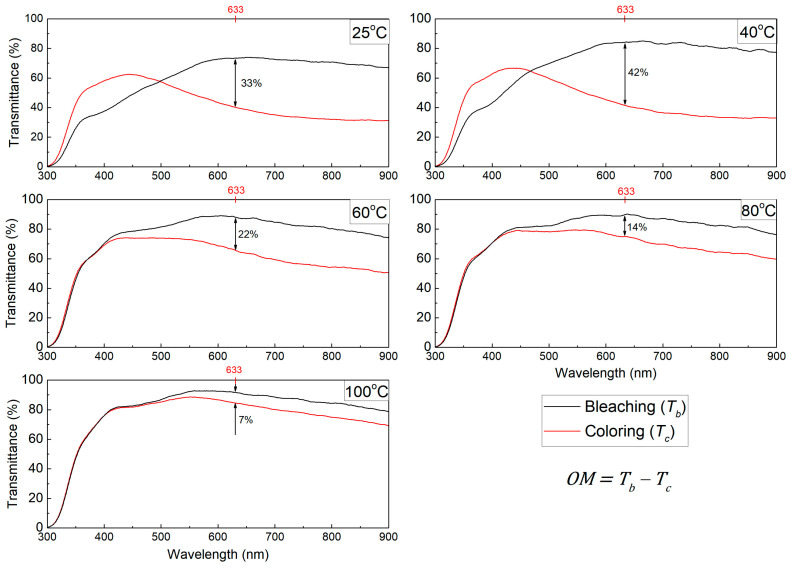
The OM graphs for all samples, highlighting the transmittance contrast between the coloring and bleaching states in the glass/ITO/WO_3_/electrolyte/ITO/glass structure.

Figure 7 depicts the *OM* calculation for all samples. As observed, the 40 °C sample showed the best *OM* whereas the 100 °C showed the lowest *OM*. This corresponds to the decrease in the ionic diffusion and the decrease in inserted charges discussed previously.

**Figure 8:**
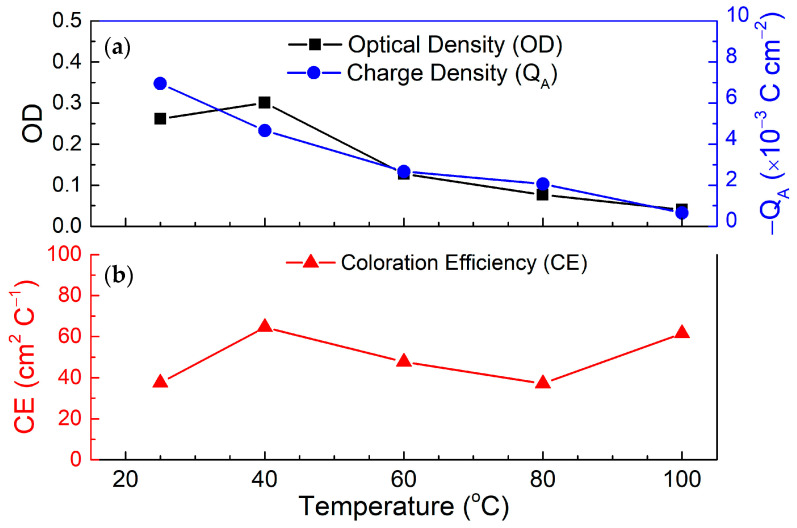
(**a**) The Δ*OD*, *Q_A_*, and (**b**) *CE* as a function of temperature for the electrolyte used in the glass/ITO/WO_3_/electrolyte/ITO/glass structure.

The coloration efficiency (*CE*), optical density (Δ*OD*), and charge transfer (*Q_A_*) were graphed and tabulated in Figure 8 and Table 3, respectively. The CE value was calculated using the following equation:

(4) $$CE=\frac{\Delta OD}{Q_{A}}$$

where *CE* is the coloration efficiency in cm^2^ C^−1^, Δ*OD* represents the variation in optical density, and *Q_A_* is the maximum surface charges obtained from the cathodic (coloring) cycle during the CA measurement (*Q_A_* in C cm^−2^).

From Figure 8 and Table 3, it can be deduced that the highest CE was found to be in the 40 and 100 °C samples. Since the Δ*OD* and *Q_A_* are functions of the equation, then if Δ*OD* increases or *Q_A_* decreases this will increase the CE as a result. The Δ*OD* and *Q_A_* reported are the highest and lowest in the 40 and 100 °C, respectively. The high Δ*OD* in the 40 °C sample is due to the better overall coloration and bleaching transmittances as concluded from Figure 7. Subsequently, the 100 °C reported the lowest *Q_A_* which affected its *CE* to a large degree.

### 3.3. Electric Impedance Spectroscopy (EIS)

**Figure 9:**
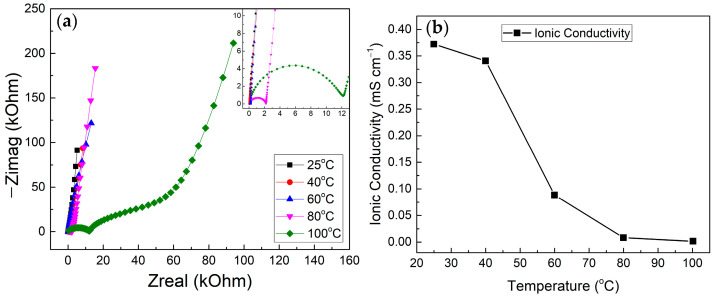
(**a**) Nyquist plots and (**b**) ionic conductivity graphs for all samples at different electrolyte fabrication temperatures. These measurements were performed on devices with the glass/ITO/electrolyte/ITO/glass structure used for EIS measurements.

The EIS measurement was conducted for all samples with a frequency range between 1 MHz and 100 mHz at an AC potential of 10 mV. Figure 9a shows the Nyquist plot for all samples. From the Nyquist plot, the ionic conductivity was calculated using the following equation:

(5) $$\sigma =\frac{l}{R\times A}$$

where *σ* stands for ionic conductivity in S cm^−1^, *l* for electrolyte thickness in cm (*l* = 0.05 cm), *R* for bulk resistance in Ω, and *A* for electrolyte effective area in cm^2^ (*A* = 2.64 cm^2^).

From Figure 9b, it can be observed that the *σ* decreased as the temperature increased. The decrease in ion conductivity can be attributed to the decrease in ion mobility due to the transitioning of the solid matrix to completely solid as more trapped solvents are being evaporated as the temperature increases [29]. Based on the distinct Nyquist plot observed in Figure 9 for the 100 °C sample, it is suggested that the near-complete evaporation of the propylene carbonate solvent has resulted in the formation of a predominantly PMMA-LiClO_4_ solid electrolyte, where the conductivity is governed by diffusion and cationic transport along the polymer chains rather than lithium−ion transport in the liquid phase.

### 3.4. ECD Stability

**Figure 10:**
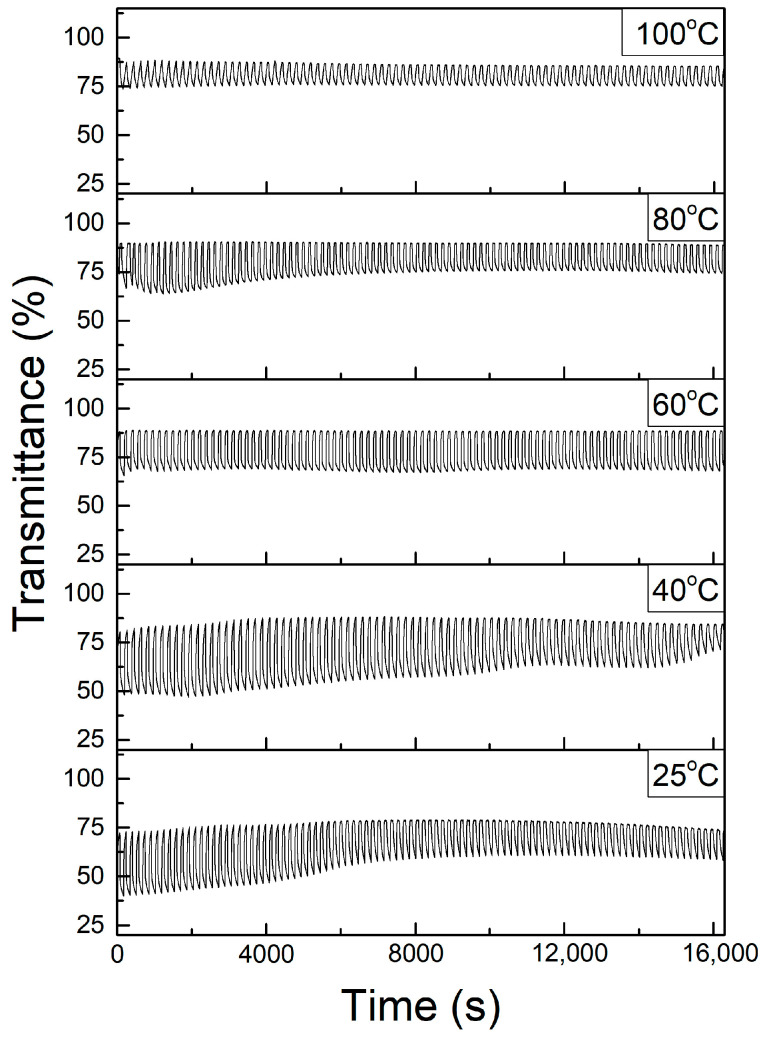
Stability test for all samples, showing coloring and bleaching transmittances during using CA for the glass/ITO/WO_3_/electrolyte/ITO/glass structure.

A stability test was conducted for all samples to determine the durability of the samples under consequent coloring and bleaching. The samples were applied to a constant voltage of −3 V (coloring) and 3 V (bleaching) for a duration of 100 s and 60 s for 100 cycles, respectively. Coloring and bleaching transmittances were recorded during the coloring and bleaching cycles. Figure 10 shows the stability graphs for all samples.

As observed from Figure 10, it can be deduced that stability increases as the temperature increases. However, as discussed before the degree of coloring degrades more as the temperature increases. This can be due to the total elimination of residual solvents as the temperature increases [29]. Furthermore, as the temperature increases the polymer matrix becomes more solid which reduces the intercalation and deintercalation of ions impact on the polymer matrix, as mentioned previously.

## 4. Conclusions

This work successfully studied the PMMA-based polymer electrolyte in electrochromic smart window devices of varying temperatures using the electrolyte solution casting method. The optical transmittance of all samples was considered satisfactory, specifically in the visible region wavelength, with a range between 80 and 100%. Furthermore, CV measurements demonstrated a slight deterioration in the *D* as the electrolyte temperature increased in all samples. The switching time obtained from CA measurement slightly increased as the temperature increased, with 25 and 100 °C having the lowest and highest switching time, respectively. Likewise, the results of the *CE* revealed a similar trend as in the switching time. Eventually, from the transmittance states profile, the *OM* deteriorated significantly with an increase in temperature, as realized from the *OM* calculations. Devices fabricated at 40 °C showed optimal *CE* and *OM*, making this temperature preferable for applications focused on the switching speed and transparency. However, higher temperatures (e.g., 80 °C and 100 °C) promoted greater stability under cycling, though at the expense of the switching speed and optical modulation. Notably, the relationship between the fabrication temperature and performance metrics was nonlinear, with increased crystallinity at higher temperatures limiting ion mobility and impacting performance.

Further studies are required for PMMA-based polymer electrolytes of varying temperatures on the electrolyte solution casting method to explore the influence of varying temperatures on optical and electrochromic characteristics. Adjusting one of the factors in the electrolyte fabrication procedure was proposed; for example, adjusting the chemical concentrations and evaluating the effect on ECD performance. Additional characterization, such as X-ray diffraction (XRD), energy-dispersive X-ray (EDX), and field emission scanning electron microscopy (FESEM), was strongly recommended to understand a further increase in the temperature effect on the ECD performance.

## Figures and Tables

**Table 1:** Anodic and cathodic diffusion for all samples at different electrolyte fabrication temperatures.

Temperature (°C)	Anodic Diffusion, *D_a_* (×10^−18^ cm^2^ s^−1^)	Cathodic Diffusion, *D_c_* (×10^−18^ cm^2^ s^−1^)
25	8.15	4.32
40	3.64	2.03
60	0.47	1.29
80	0.063	0.11
100	0.0054	0.0060

**Table 2:** Switching times for all samples at different electrolyte fabrication temperatures.

Temperature (°C)	Coloration Time (s)	Bleaching Time (s)
25	1	1.4
40	0.6	1.3
60	1.2	2.5
80	11.3	20.5
100	22.1	27.1

**Table 3:** The *CE*, Δ*OD*, and *Q_A_* for all samples at different electrolyte fabrication temperatures.

Temperature (°C)	Coloration Efficiency, *CE* (cm^2^ C^−1^)	Optical Density, Δ*OD*	Surface Charge, *Q_A_* (×10^−3^ C cm^−2^)
25	37.53	0.26	−0.0070
40	64.54	0.30	−0.0047
60	47.71	0.13	−0.0027
80	37.14	0.077	−0.0021
100	61.58	0.040	−0.00065

## Data Availability

The data that support the findings of this study are available on request.
